# Supplementary figures and images for: The Streptochaeta Genome and the Evolution of the Grasses
Source: Front Plant Sci. 2021 Oct 4;12:710383. doi: 10.3389/fpls.2021.710383 (PMC8521107; doi:10.3389/fpls.2021.710383)

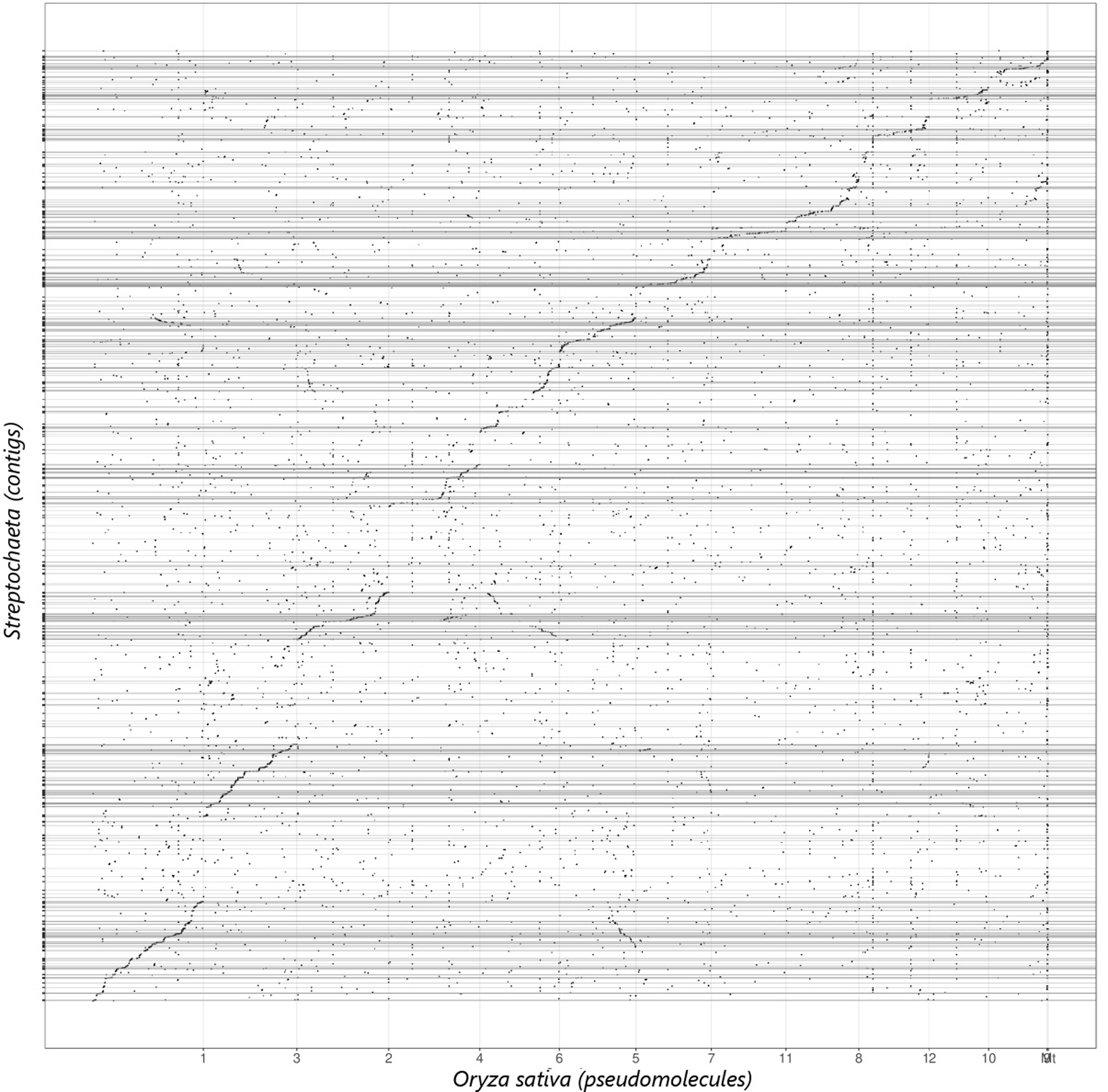

Supplement: Supplementary Figure 1 — Dot plots depicting whole genome alignments of Streptochaeta scaffolds with rice chromosomes. Dots aligned diagonally shows conserved synteny between these genomes. [file Image_1.JPEG]

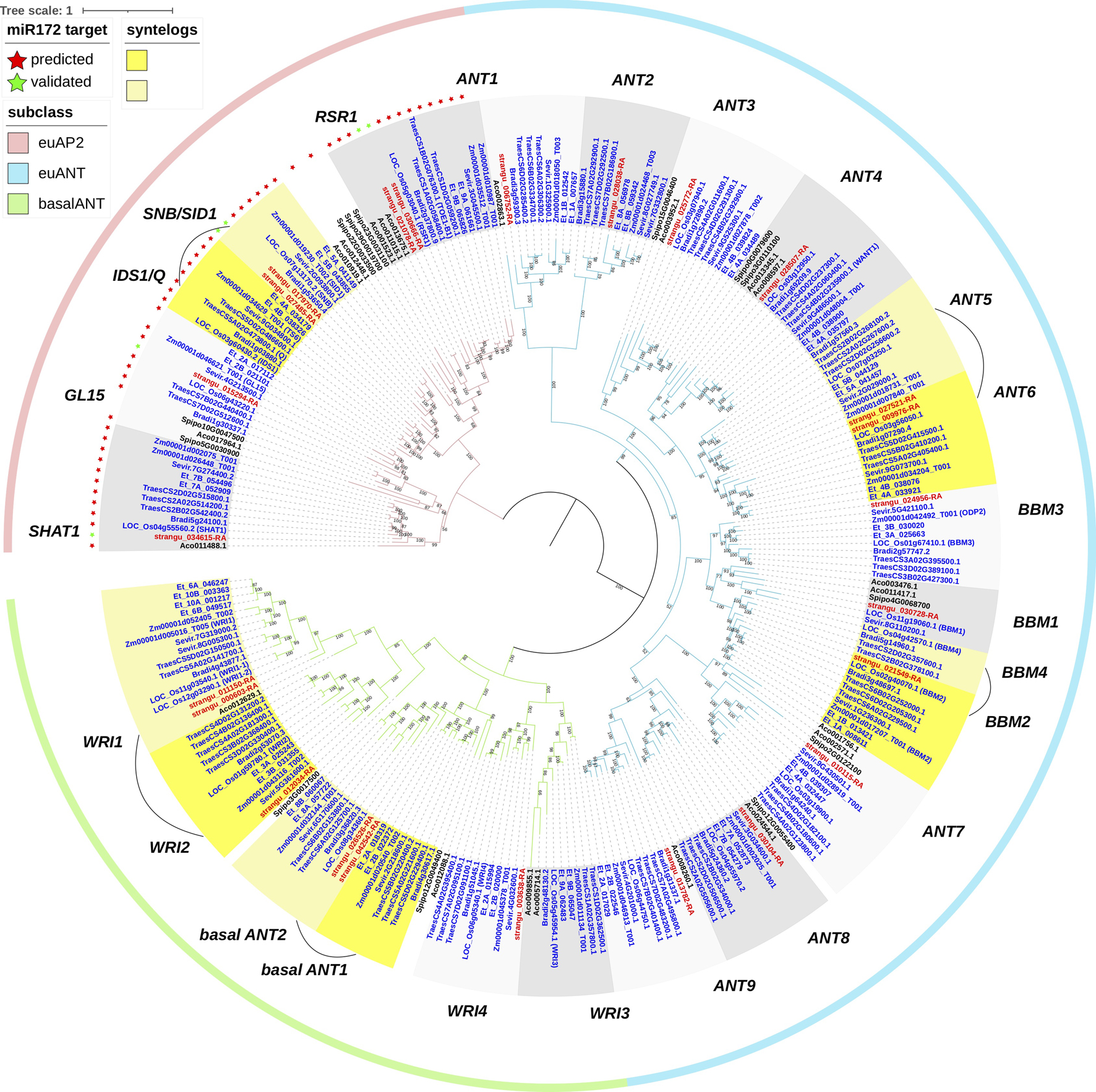

Supplement: Supplementary Figure 2 — Maximum likelihood tree of AP2-like genes with gene names. Bootstrap values are shown on the branches. Each subclade is shaded in two gray colors and named either by known genes within the subclade or subfamily name with a number. Subclades with syntenic genes in Brachypodium, Oryza, or Setaria are shaded in two colors of yellow, and syntenic pairs are connected by an arc. Predicted and experimentally validated miR172 binding sites are denoted by red and green stars, respectively. [file Image_2.JPEG]

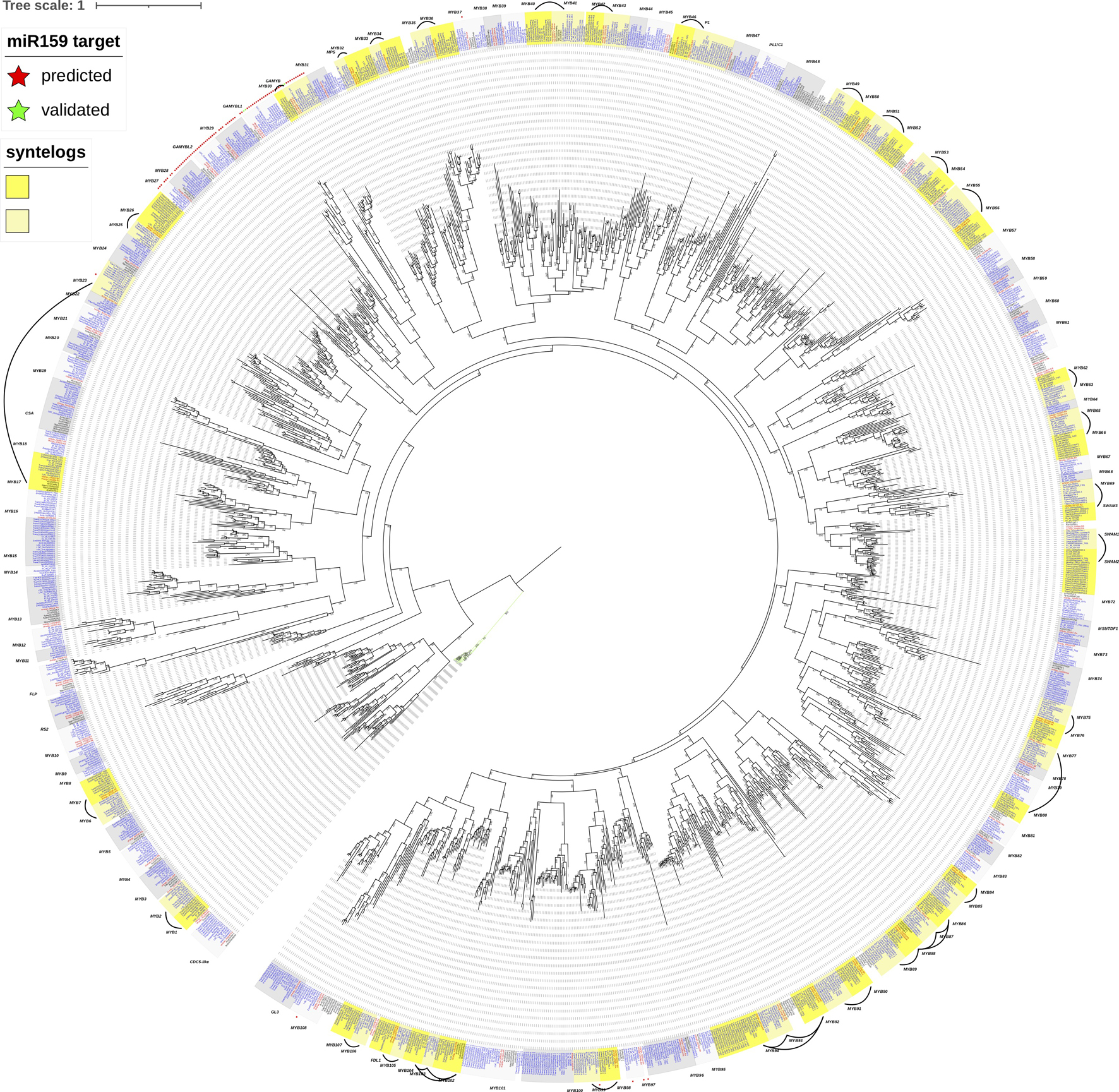

Supplement: Supplementary Figure 3 — Maximum likelihood tree of R2R3 genes with gene names. Bootstrap values are shown on the branches. Each subclade is shaded in two gray colors and named either by known genes within the subclade or subfamily name with a number. Subclades with syntenic genes in Brachypodium, Oryza, or Setaria are shaded in two colors of yellow, and syntenic pairs are connected by an arc. Predicted and experimental validated miR159 binding sites are denoted by red and green stars, respectively. [file Image_3.JPEG]
